# Seneca Valley virus infection exploits DNA damage response to facilitate viral replication

**DOI:** 10.1128/jvi.02211-24

**Published:** 2025-02-26

**Authors:** Jiangwei Song, Zijian Li, Jingjing Yang, Ruiyi Ma, Dan Wang, Rong Quan, Xuexia Wen, Jue Liu

**Affiliations:** 1 Beijing Key Laboratory for Prevention and Control of Infectious Diseases in Livestock and Poultry, Institute of Animal Husbandry and Veterinary Medicine, Beijing Academy of Agriculture and Forestry Sciences 656308 , Beijing, China; 2 College of Animal Science and Veterinary Medicine, Shenyang Agricultural University 98428 , Shenyang, China; 3 College of Veterinary Medicine, Yangzhou University 614704 , Yangzhou, China; University of Kentucky College of Medicine, Lexington, Kentucky, USA

**Keywords:** Seneca Valley virus (SVV), DNA damage response (DDR), DNA repair, DNA double-strand break (DSB), viral replication

## Abstract

Seneca Valley virus (SVV) is an emerging pathogen that causes severe vesicular diseases in swine, posing a significant threat to the global pork industry. DNA and RNA viruses manipulate the host DNA damage response (DDR) to modulate cellular machinery and facilitate their life cycles. However, the interaction between the host DDR and SVV infection remains unexplored. Here, we aimed to comprehensively investigate the DDR and DNA repair signaling pathways during SVV infection. We found that SVV infection causes DNA damage and triggers distinct DDR signaling pathways, including ataxia telangiectasia-mutated (ATM) kinase, ATM-Rad3-related kinase, and DNA-dependent protein kinase. However, it failed to induce the formation of γH2AX and 53BP1 foci, resulting in unrepaired DNA damage. Furthermore, we found that SVV 2B and 2C proteins can activate DDR signaling pathways and impair DNA repair. SVV-induced DDR triggered NF-κB signaling accompanied by upregulation of pro-inflammatory cytokines, as evidenced by the inhibition of ATM kinase, abolished SVV-induced NF-κB activation. Inhibition of the ATM pathway attenuated SVV replication. These findings expand our understanding of host DDR manipulation during viral infection and provide crucial insights into a novel mechanism exploited by SVV to regulate the inflammatory response for efficient replication.

**IMPORTANCE:**

DDR is a cellular machinery that senses and repairs host DNA lesions to maintain genome integrity. Viruses have evolved diverse strategies to manipulate host DDR for replicative efficiency. SVV is an emerging virus that causes vesicular diseases in pigs and severely threatens the swine industry. However, the interaction between SVV and DDR remains unclear. Here, we found that SVV modulates host DDR pathways to facilitate viral replication. Our results demonstrated that SVV infection causes DNA damage, activates ATM-mediated DNA double-strand break response, and impedes DNA repair. SVV 2B and 2C proteins induced DNA damage and activated the DDR pathway while impairing repair mechanisms. This study revealed a fine-tuned molecular mechanism of SVV-modulated DDR that contributes to viral replication, facilitating deeper insight into SVV replication.

## INTRODUCTION

Seneca Valley virus (SVV), first discovered in PER.C6 cells in the United States in 2002 (1, 2), is an oncolytic picornavirus that selectively infects and lyses cancer cells. Clinical trials have confirmed its potential as a human cancer treatment (3, 4). SVV causes typical vesicular lesions in pigs, with clinical symptoms similar to those of foot-and-mouth disease virus, vesicular stomatitis virus, and swine vesicular disease virus.

SVV is a non-enveloped, positive-stranded RNA virus from the *Picornaviridae* family (1, 5). The genome is approximately 7.2 kb in length comprising one open reading frame (ORF), which translates into a polyprotein precursor that is subsequently processed into structural and non-structural proteins, including the leader (L) protein, P1 (VP4, VP2, VP3, and VP1), P2 (2A, 2 B, and 2C), and P3 (3A, 3 B, 3C, and 3D) (1). SVV 3C protease (3C^pro^) antagonizes host innate immune responses and selective autophagy by targeting key molecules for cleavage and degradation, such as mitochondrial antiviral signaling protein (6), signal transducer and activator of transcription (STAT) 1/STAT2 (7), interferon regulatory factor (IRF)3/IRF7/IRF9 (7, 8), selective autophagy receptor SQSTM1/p62, and optineurin (9, 10). SVV 2B induces mitochondrial damage, releasing mitochondrial DNA into the cytoplasm, where it binds to cyclic GMP-AMP synthase (cGAS) and activates the cGAS-mediated type I interferon (IFN) response (11). SVV 2C inhibits type I IFN production by degrading cGAS and retinoic acid-inducible gene I (11, 12).

The DNA damage response (DDR) is a sophisticated surveillance network that safeguards genomic integrity (13, 14). It can be activated by viral infections, leading to cell cycle checkpoint activation, DNA repair, senescence, inflammation, or apoptosis (15–17). Following DNA damage, a network of pathways is rapidly triggered to sense DNA lesions (18, 19). The DDR signaling pathway is activated by three related phosphatidylinositol 3-kinase-like kinases: ataxia telangiectasia-mutated (ATM) kinase, ATM-Rad3-related kinase (ATR), and DNA-dependent protein kinase (DNA-PK) (20). DNA double-strand breaks (DSBs) and single-strand breaks are detected using the Mre11-Rad50-NBS1 (MRN) complex and replication protein A (RPA), which guide the recruitment of ATM and ATR, respectively. ATM and ATR undergo autophosphorylation and subsequently phosphorylate various DDR factors, such as effector kinases checkpoint kinase-1 (CHK1) and CHK2, which are crucial for inducing cell cycle arrest (4). DNA-PK is activated in response to DSBs and is involved in DNA repair via a non-homologous end-joining (NHEJ) pathway (21, 22). Homologous recombination and NHEJ are the two major pathways to repair DSBs (23, 24). The NHEJ-mediated repair pathway is mainly regulated by p53-binding protein 1 (53BP1), a chromatin-binding protein that regulates DSB repair (25, 26). Upon DSBs, 53BP1 is recruited to damaged DNA sites where nuclear foci are formed and transduce DNA repair processes (20).

Numerous viruses can trigger the DDR in host cells during their life cycles and consequently develop various strategies to exploit and control DDR pathways for their benefit (27). Studies have indicated that viruses manipulate the early stages of DDR pathways to ensure successful genome replication while dampening downstream effects to prevent adverse outcomes of pathway activation (27). DDRs are involved in the replication of DNA and RNA viruses. DNA viruses, such as human bocavirus 1 (HBoV1) infection, induce a DDR with activation of ATM, ATR, and DNA-PKcs (28, 29). Adeno-associated virus DNA replication initiates cellular DDR, leading to DNA repair that contributes to viral DNA replication (30). Kaposi’s sarcoma herpesvirus (KSHV) exploits DDR kinases ATM and DNA-PKcs for genome circularization and latency establishment (31). For RNA viruses, severe acute respiratory syndrome coronavirus 2 (SARS-CoV-2) infection activates DDR, inducing inflammation and cellular senescence (32). ATM-mediated DSB response facilitates syncytium formation and promotes Newcastle disease virus replication (33). The ATM-CHK2 axis-mediated DSB pathway is crucial for chikungunya virus and hepatitis C virus (HCV) infections (34, 35). Notably, DNA damage is sensed by the innate immune system, which involves the activation of NF-κB and cGAS-STING signaling pathways (36–40). However, the role of DDR during SVV infection remains elusive, and whether SVV manipulates the host DDR mechanisms for replication remains unknown.

This study aimed to comprehensively investigate the DDR and DNA repair signaling pathways during SVV infection. Our findings reveal the crucial role of ATM-mediated DSBs in SVV replication and provide novel insights into SVV pathogenesis mechanisms.

## RESULTS

### SVV infection causes DNA damage and a distinctive DDR activation

**Fig 1:**
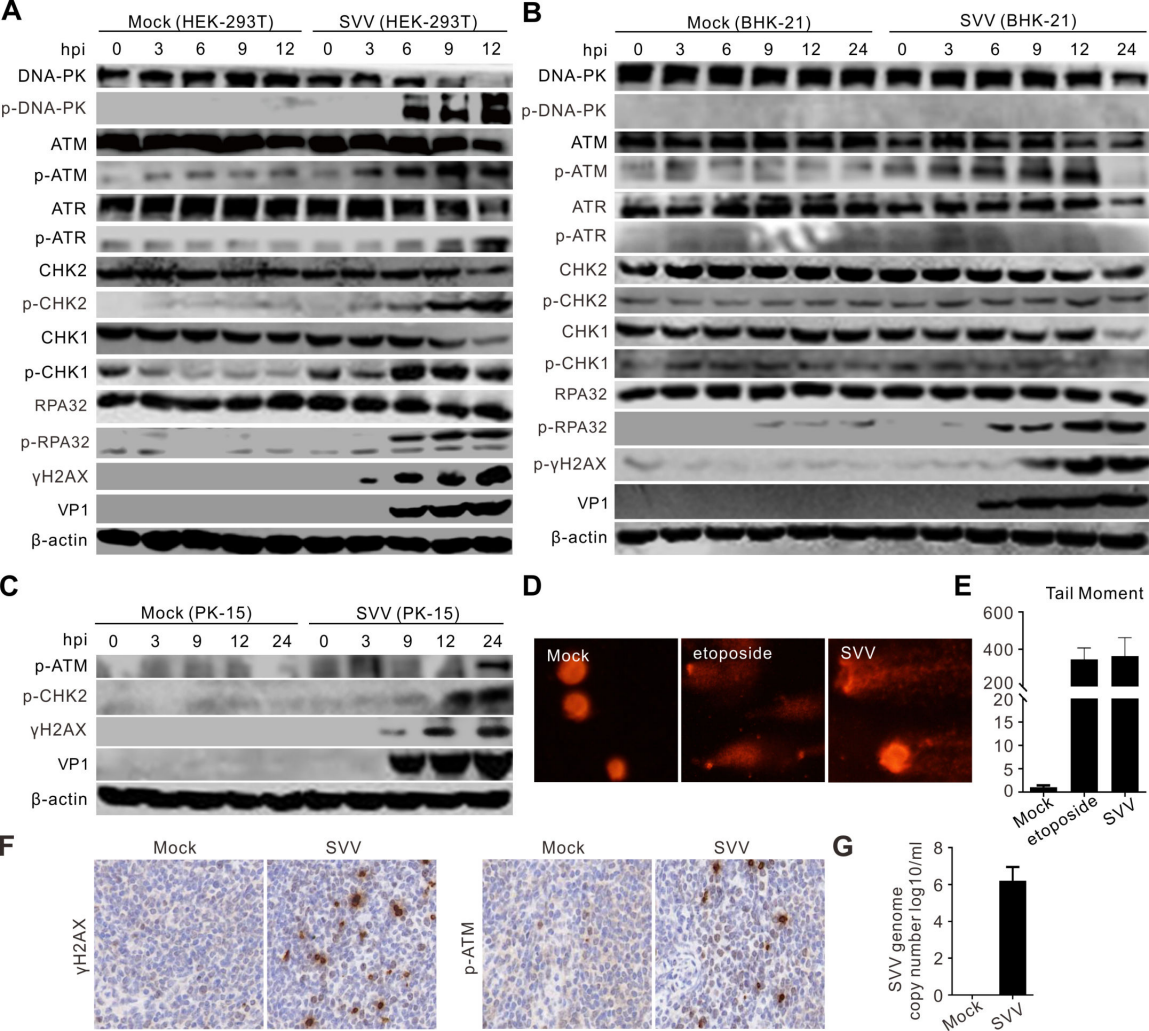
SVV infection induced DNA damage and activated DDR. (**A–C**) HEK-293T cells, BHK-21 cells, and PK-15 cells were infected with SVV (multiplicity of infection [MOI] = 5), respectively. The cell lysates were collected at indicated times and analyzed by immunoblotting with the indicated antibodies, with β-actin as an internal control. (**D**) BHK-21 cells were infected with SVV (MOI = 5) for 12 h or treated with etoposide. Cells (1 × 10^5^) per treatment were collected for the alkaline comet assay. Samples were analyzed by fluorescence microscope. (**E**) The degree of DNA damage was indicated by DNA percentage in the tail using OpenComet software. (**F**) Immunohistochemical staining of spleen tissues of SVV-inoculated or mock-inoculated piglets for determination of γH2AX and p-ATM expression (brown signals) using mouse monoclonal antibodies against γH2AX (sc-517348, Santa Cruz) and p-ATM (sc-47739, Santa Cruz), respectively. (**G**) Quantification of SVV RNA by quantitative reverse transcription-PCR. Spleens were collected on day 10 post-infection, and levels of SVV RNA were expressed as log10 genome copy number per milliliter.

**Fig 2:**
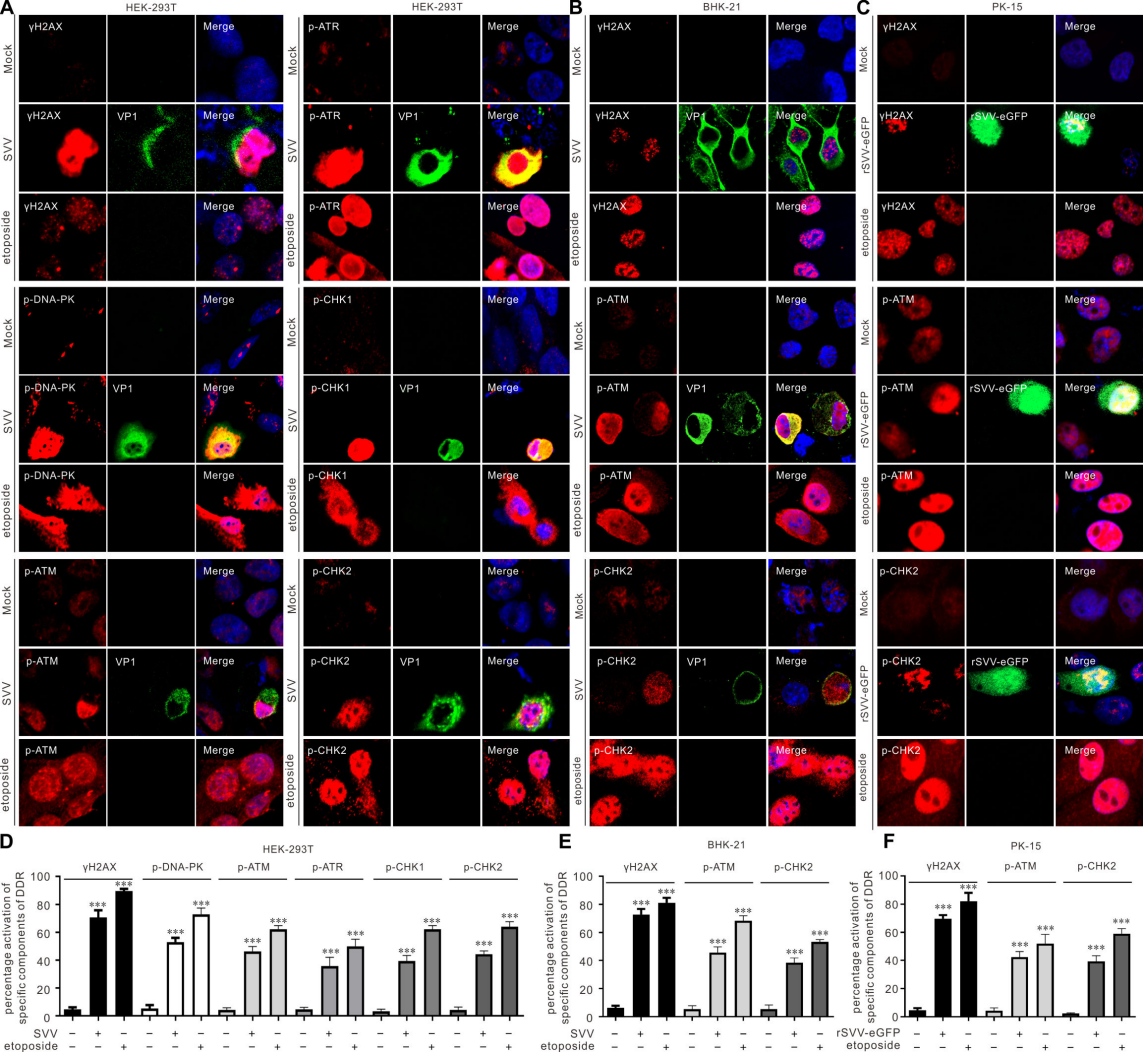
SVV infection induced ATM-CHK2 pathway in different host cells. (**A–C**) HEK-293T cells, BHK-21 cells, and PK-15 cells were infected with SVV or eGFP-tagged recombinant SVV (rSVV-eGFP) (MOI = 1), respectively. Cells were stained with the indicated antibody (red), VP1 antibody (green), and 4',6-diamidino-2-phenylindole (blue), then examined by confocal microscopy. (**D–F**) Statistical analysis was performed for phosphorylated DDR components after SVV infection or treated with etoposide from (**A–C**). ***P < 0.001.

We observed that SVV infection triggered autophosphorylation of DNA-PK (p-DNA-PKcs, S2056), ATM (p-ATM, S1981), and ATR (p-ATR, T1989) in HEK-293T cells (Fig. 1A). The downstream target of ATM, CHK2 (p-CHK2, T68), and the downstream of ATR, CHK1, were phosphorylated (p-CHK1, S345) after SVV infection (Fig. 1A). The phosphorylation of ATR downstream effector replication protein A32 (p-RPA32, S33) and the phosphorylation of H2A histone family member X (γH2AX, S139) are hallmarks of the DDR. SVV infection induced p-RPA32 and γH2AX phosphorylation in HEK-293T cells (Fig. 1A). Conversely, the DNA-PK and ATR pathways did not respond to SVV infection in either BHK-21 (Fig. 1B) or PK-15 cells (Fig. 1C), highlighting the potential mechanistic differences between cell types in response to DDR signaling. The results were reproduced *in vivo*, as demonstrated by the increased expression of γH2AX and p-ATM in the spleens of piglets infected with SVV through immunohistochemical assays (Fig. 1F and G). Comet assay was performed to assess the effects of SVV infection on DNA integrity in BHK-21 cells. Using etoposide as a positive control to induce DDR, we discovered that SVV infection or etoposide treatment induced DNA fragmentation (Fig. 1D), as measured by the tail moment (Fig. 1E). This indicates that SVV infection led to an increase in the percentage of tail DNA (comet tail), which served as evidence of cellular DNA damage. We observed no DNA migration in mock-infected cells (Fig. 1D and E). Next, we evaluated SVV-activated DDR by immunostaining with phosphorylated antibodies against components of DDR pathways. In etoposide-treated cells, p-DNA-PKcs, p-ATM, γH2AX, and p-RPA32 expressions were significantly increased (Fig. 2A and D). Similarly, as shown in Fig. 2A, the levels of p-DNA-PKcs, p-ATM, p-ATR, γH2AX, and p-RPA32 were significantly induced after SVV infection in HEK-293T cells. Conversely, SVV infection activated the ATM-CHK2 pathway in the BHK-21 (Fig. 2B and E) and PK-15 cells (Fig. 2C and F). Our data indicate that SVV infection leads to DNA damage and induces distinct DDR activation, depending on the cell type.

### SVV 2B and 2C contribute to DDR pathway activation

**Fig 3:**
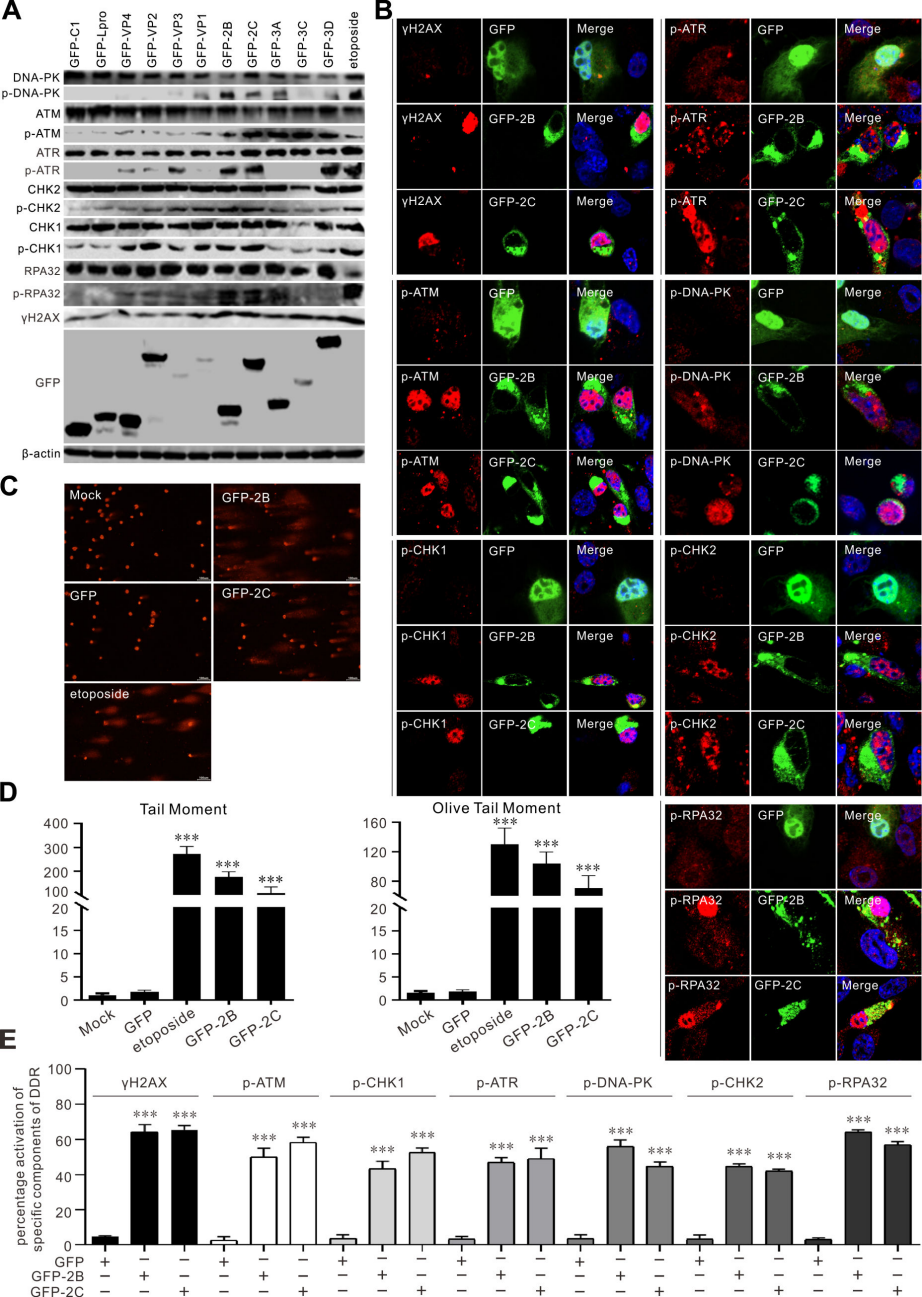
SVV 2B and 2C were sufficient to induce DDR and activate ATM, ATR, and DNA-PKcs. (**A**) HEK-293T cells were transfected with GFP-tagged SVV protein expression plasmids for 24 h. Cell samples were subjected to immunoblotting with the indicated antibodies. (**B**) HEK-293T cells were transfected with GFP empty vector, GFP-2B, or GFP-2C for 24 h. Cells were stained with the indicated antibodies (red) and DAPI (blue), then examined by confocal microscopy. (**C**) HEK-293T cells were transfected with GFP empty vector, GFP-2B, and GFP-2C or treated with etoposide (10 µM) for 24 h. Cells (1 × 10^5^) per treatment were collected for the alkaline comet assay. Samples were analyzed by fluorescence microscope. (**D**) The degree of DNA damage was indicated by DNA percentage in the tail using OpenComet software. ***P < 0.001. (**E**) Statistical analysis was performed for phosphorylated DDR components after transfection with GFP empty vector, GFP-2B, and GFP-2C from panel** B**. ***P < 0.001.

To examine which viral proteins are responsible for DDR activation, we individually overexpressed SVV proteins. Among these viral protein products, proteins 2B and 2C are critical for DDR induction. In contrast to etoposide treatment, Western blotting revealed that their sole expression was sufficient to induce ATM-CHK2, ATR-CHK1, DNA-PK_CS_, γH2AX, and RPA32 phosphorylation (Fig. 3A). 3D also notably activates ATM and ATR pathways and slightly activates the DNA-PKcs pathway (Fig. 3A). Additionally, we found that 3C^pro^ expression reduced the expression of DNA-PK_CS_, CHK1, CHK2, and RPA32 (Fig. 3A). Consistent with this, phosphorylation was also detected by immunofluorescence, and the fluorescence intensity of γH2AX, p-RPA32, p-ATM, p-CHK2, p-ATR, p-CHK1, and p-DNA-PKcs was robustly enhanced upon SVV 2B and 2C protein expression (Fig. 3B and E). DNA damage accumulation was further examined by comet assay; the expression of 2B or 2C or etoposide treatment induced DNA fragmentation (Fig. 3C and D). Collectively, these results indicate that ectopic expression of SVV 2B or 2C protein is sufficient to induce DDR and activate the expression of γH2AX and p-RPA32, along with the activation of ATM-CHK2, ATR-CHK1, and DNA-PKcs.

### SVV infection degraded MRN components but did not prevent DDR signaling

**Fig 4:**
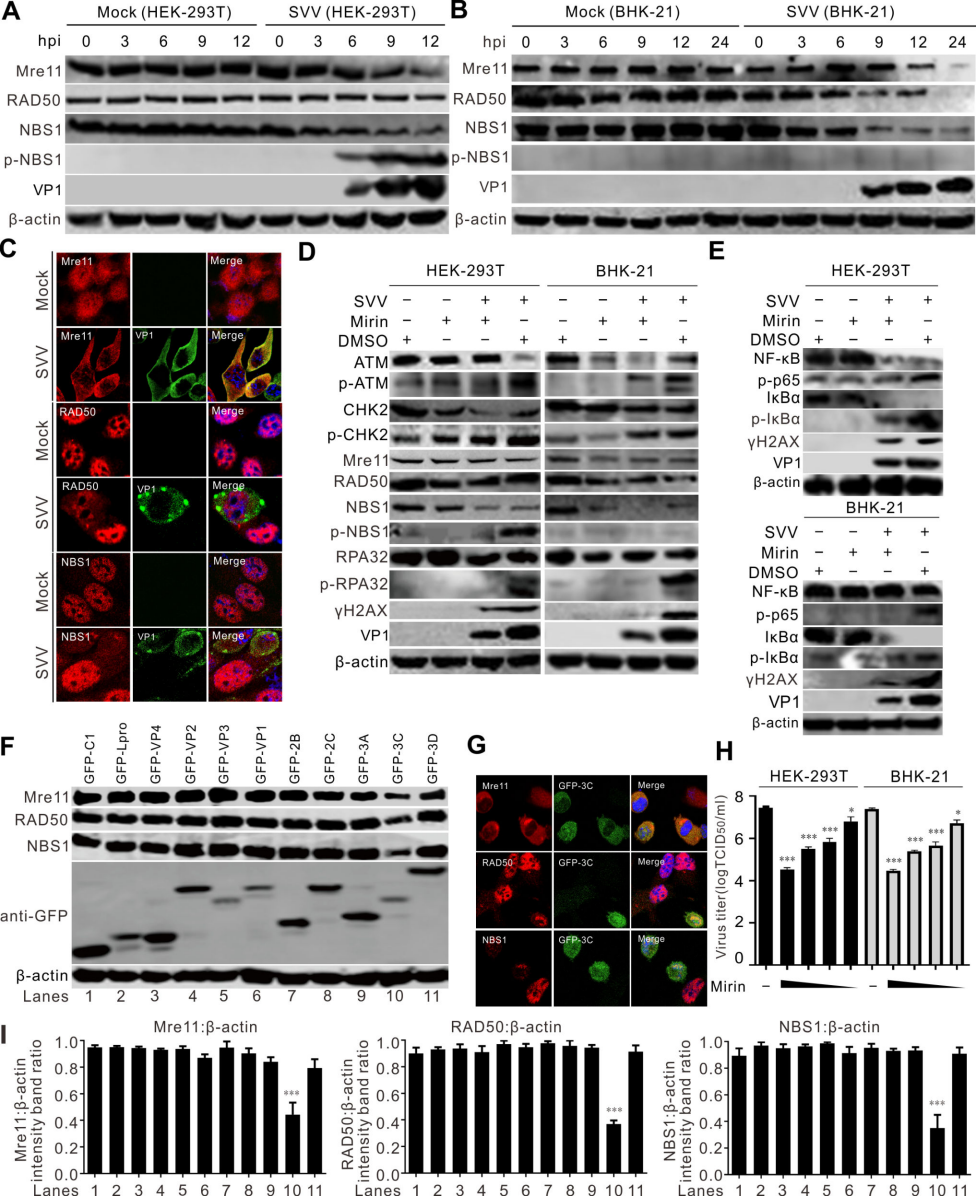
SVV degrades MRN components but do not prevent DDR signaling. (**A and B**) HEK-293T cells and BHK-21 cells were infected with SVV (MOI = 5), respectively. The cell lysates were collected at indicated times and analyzed by immunoblotting with the indicated antibodies. (**C**) BHK-21 cells were infected with SVV (MOI = 1). Cells were stained with Mre11 (red), RAD50 (red), and NBS1 antibody (red), VP1 monoclonal antibody (green), and DAPI (blue), then examined by confocal microscopy. (**D and E**) BHK-21 cells were infected with SVV (MOI = 5) and treated with mirin (10 µM). The cell lysates were collected at 12 hpi and analyzed by immunoblotting with the indicated antibodies. (**F**) BHK-21 cells were transfected with different green fluorescent protein (GFP)-tagged SVV protein expression plasmids for 24 h. Cell samples were subjected to immunoblotting with the indicated antibodies. (**G**) BHK-21 cells were transfected with GFP-3C for 24 h; cells were stained with Mre11 (red), RAD50 (red), and NBS1 antibody (red) and DAPI (blue), then examined by confocal microscopy. (**H**) Viral titers of SVV after treatment with mirin (20, 10, 5, or 1 µM) with various concentrations in BHK-21 cells, respectively. At 12 hpi, the total viruses were titrated with the TCID_50_ assay. (**I**) The ratios of β-actin from three independent experiments depicted in panel F are shown. ImageJ was used to quantify the level of proteins. ***P < 0.001, *P < 0.05.

**Fig 5:**
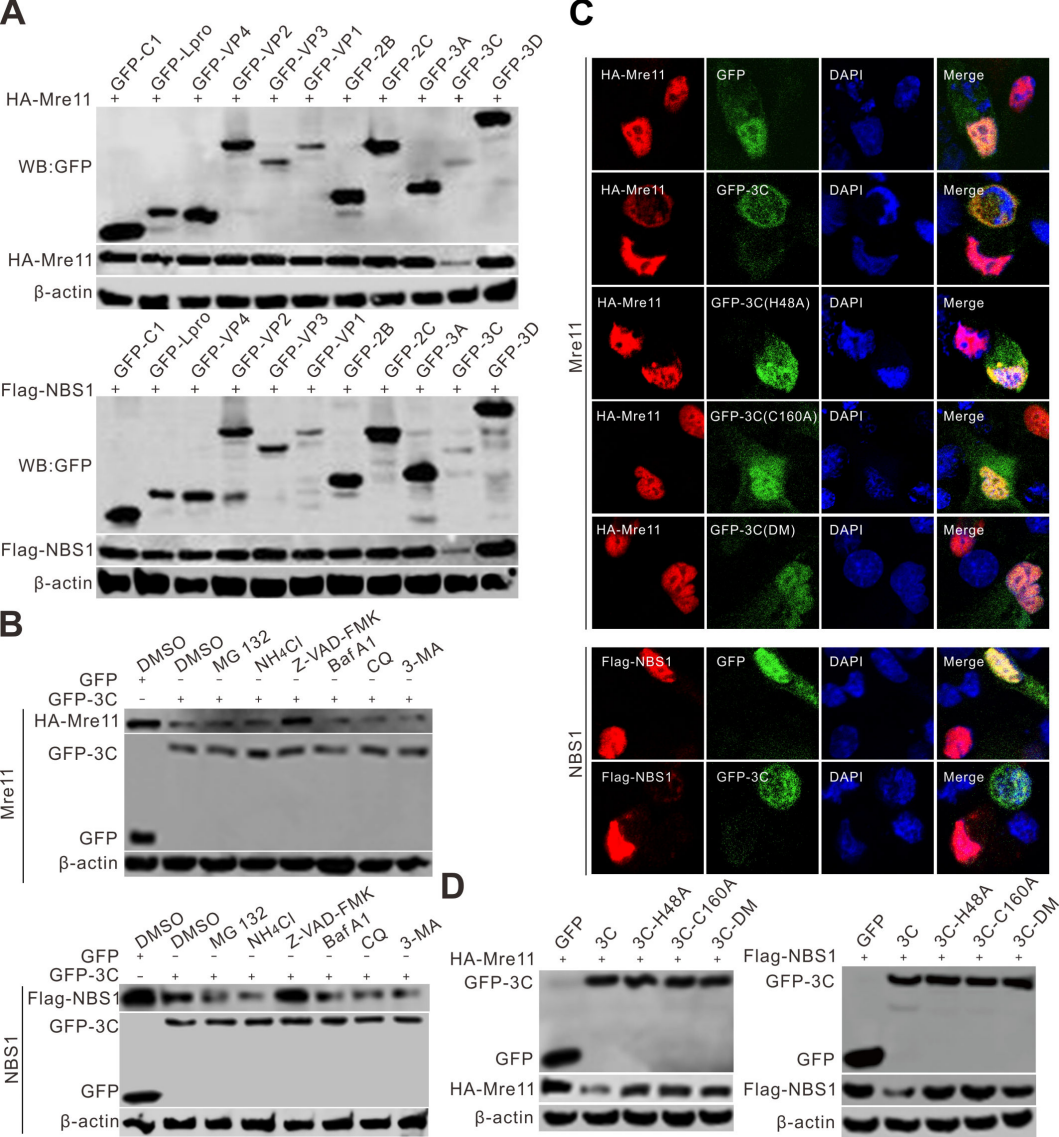
SVV 3C^pro^ induced Mre11 and NBS1 degradation via the caspase pathway. (**A**) BHK-21 cells were co-transfected with HA-Mre11 or Flag-NBS1 with GFP-viral gene plasmids for 24 h, then the samples were subjected to Western blotting analysis. (**B**) BHK-21 cells were transfected with GFP empty vector or GFP-3C with HA-Mre11 and Flag-NBS1, respectively. At 16 hpt, cells were treated with MG132 (10 µM), Z-VAD-FMK (50 µM), NH_4_Cl (10 mM), CQ (40 µM), Baf A1 (200 nM), and 3-MA (25 mM) for 12 h. The samples were subjected to Western blotting. (**C**) BHK-21 cells were co-transfected GFP, GFP-3C, GFP-3C-H48A, GFP-3C-C160A, and GFP-3C-DM (H48AC160A) with HA-Mre11 or Flag-NBS1. At 24 h post-transfection, the samples were subjected to immunofluorescence analysis with the indicated antibodies then examined by confocal microscopy. (**D**) BHK-21 cells were co-transfected with GFP-3C and its protease activity mutants, GFP-3C (H48A), GFP-3C (C160A), and GFP-3C (DM) (H48A and C160A double mutation), together with HA-Mre11 and Flag-NBS1, respectively. At 24 hpt, the samples were subjected to Western blotting. DMSO, dimethyl sulfoxide.

Recognition of a DSB by MRN leads to the activation of ATM kinase and subsequent CHK2 phosphorylation. NBS1 phosphorylation suggests the potential initiation of a DSB response. Next, we determined whether SVV infection activated MRN complexes. Fig. 4A and B show the phosphorylation of NBS1 in SVV-infected HEK-293T cells but not in BHK-21 cells. SVV infection inactivated MRN complexes, as reflected by the degradation of Mre11 and NBS1 (Fig. 4A through C) and mislocalization of Mre11, causing its accumulation in the cytoplasm (Fig. 4C). Mirin, an MRN complex inhibitor, prevented SVV infection-induced global phosphorylation of DDR substrates (Fig. 4D) and inhibited the activation of the ATM-CHK2 pathway during SVV replication (Fig. 4D). Mirin treatment notably impaired SVV infection and induced NBS1 phosphorylation, suggesting that it inhibits the MRN pathway (Fig. 4D). Additionally, mirin treatment dampened the SVV-induced ATM-CHK2 signaling pathway, as indicated by the phosphorylation of ATM, CHK2, and H2AX, suggesting that MRN leads to the activation of ATM kinase (Fig. 4D). Mirin significantly reduced the replication of SVV, resulting in weakened SVV-induced NF-κB phosphorylation (Fig. 4E). We found that SVV 3C^pro^ was responsible for the degradation of Mre11 and NBS1 (Fig. 4F and G) and the accumulation of Mre11 in the cytoplasm (Fig. 4G). In the presence of mirin, the viral titers were significantly reduced in a dose-dependent manner (Fig. 4H). The degradation of NBS1 and Mre11 induced by 3C^pro^ via the caspase pathway (Fig. 5A and B) depended on the protease activity (Fig. 5D). In the presence of GFP-3C, Mre11 was relocated from the nucleus and then redistributed into the cytoplasm (Fig. 5C). On the contrary, the GFP-3C mutants lacking protease activity exerted no influence on the distribution of Mre11 (Fig. 5C). Collectively, these findings indicate that SVV infection inactivates the MRN but does not prevent ATM-Chk2 axis activation.

### SVV 2B and 2C impair 53BP1 recruitment at DSBs and hamper DNA repair

**Fig 6:**
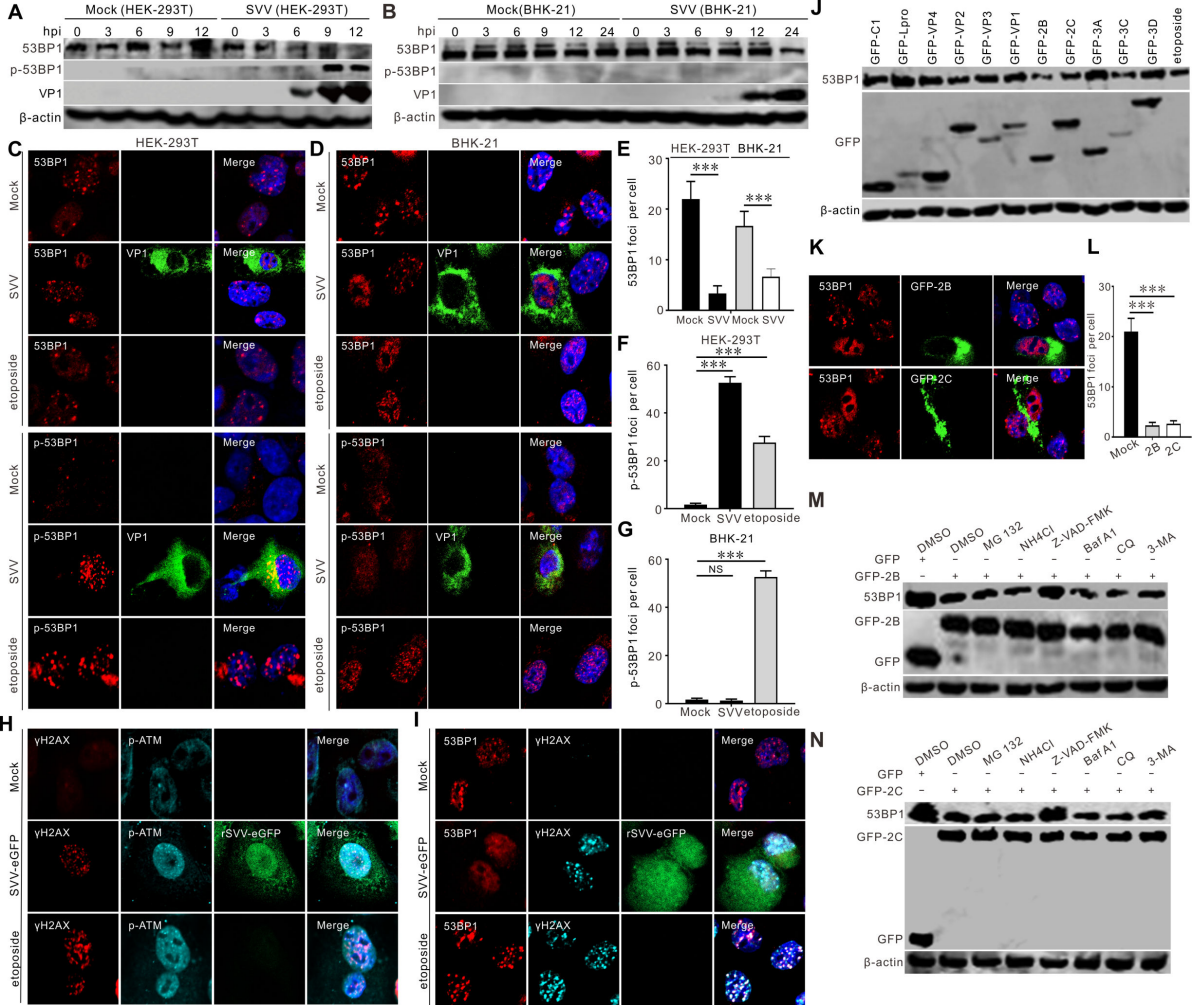
SVV 2B and 2C protein impairs DNA repair. (**A and B**) HEK-293T cells and BHK-21 cells were infected with SVV (MOI = 5), respectively. The cell lysates were collected at indicated times and analyzed by immunoblotting with indicated antibodies. (**C and D**) HEK-293T cells and BHK-21 cells were infected with SVV (MOI = 1) or treated with etoposide (10 µM), respectively. Cells were stained with 53BP1 (red), p-53BP1 antibody (red), VP1 monoclonal antibody (green), and DAPI (blue), then examined by confocal microscopy. (**E**) Quantification of 53BP1 foci shown in panels** C and D**. Each dot represents the number of 53BP1 foci per nucleus. (**F and G**) Quantification of p-53BP1 foci shown in panels** C and D**, respectively. Each dot represents the number of p-53BP1 foci per nucleus. (**H and I**) BHK-21 cells were infected with SVV (MOI = 1) or treated with etoposide (10 µM), respectively. Cells were stained with 53BP1 (red), p-53BP1 antibody (red), and DAPI (blue), then examined by confocal microscopy. (**J**) BHK-21 cells were transfected with GFP-tagged SVV protein expression plasmids for 24 h, respectively. Cell samples were subjected to immunoblotting with indicated antibodies. (**K**) BHK-21 cells were transfected with GFP-2B or GFP-2C for 24 h. Cells were stained with 53BP1 antibody (red) and DAPI (blue), then examined by confocal microscopy. (**L**) Quantification of 53BP1 foci shown in panel** K**. The histograms indicate the number of 53BP1 foci in cells expressing GFP-2B and GFP-2C. (**M**). BHK-21 cells were transfected with GFP empty vector or GFP-2B, respectively. At 16 hpt, cells were treated with MG132 (10 µM), Z-VAD-FMK (50 µM), NH_4_Cl (10 mM), CQ (40 µM), Baf A1 (200 nM), and 3-MA (25 mM) for 12 h. The samples were subjected to Western blotting with antibody raised against 53BP1. (**N**) BHK-21 cells were transfected with GFP empty vector or GFP-2C, respectively. At 16 hpt, cells were treated with MG132 (10 µM), Z-VAD-FMK (50 µM), NH_4_Cl (10 mM), CQ (40 µM), Baf A1 (200 nM), and 3-MA (25 mM) for 12 h. The samples were subjected to Western blotting with antibody raised against 53BP1. NS, not significant, ***P < 0.001.

ATM and 53BP1 are key transducers and mediators of DSB signaling, respectively. We observed SVV-induced phosphorylation of 53BP1 in HEK-293T cells (Fig. 6A, C and F) but not in BHK-21 cells (Fig. 6B, D and G) and also noted reduced 53BP1 protein levels (Fig. 6A and B). The number of 53BP1 foci was markedly lower in SVV-infected cells than in mock-infected cells (Fig. 6C and D). Etoposide treatment increased the number of 53BP1 and γH2AX foci, p-ATM, and γH2AX co-localization per cell (Fig. 6H and I). eGFP-tagged recombinant SVV (rSVV-eGFP) infection-induced γH2AX foci accumulation was accompanied by co-localization with p-ATM in nuclear foci (Fig. 6H), while γH2AX foci accumulation was not accompanied by co-localizing 53BP1 foci in rSVV-eGFP infected BHK-21 cells (Fig. 6I). These results indicate that SVV infection impaired 53BP1 recruitment and hindered NHEJ. We confirmed that SVV 2B and 2C proteins were responsible for degrading 53BP1 (Fig. 6J) via the caspase pathway (Fig. 6M and N) and significantly decreased 53BP1 foci (Fig. 6K and L). These results demonstrated that SVV 2B and 2C proteins inhibited 53BP1 foci formation, hampering DNA repair through NHEJ.

### SVV-induced micronuclei formation

**Fig 7:**
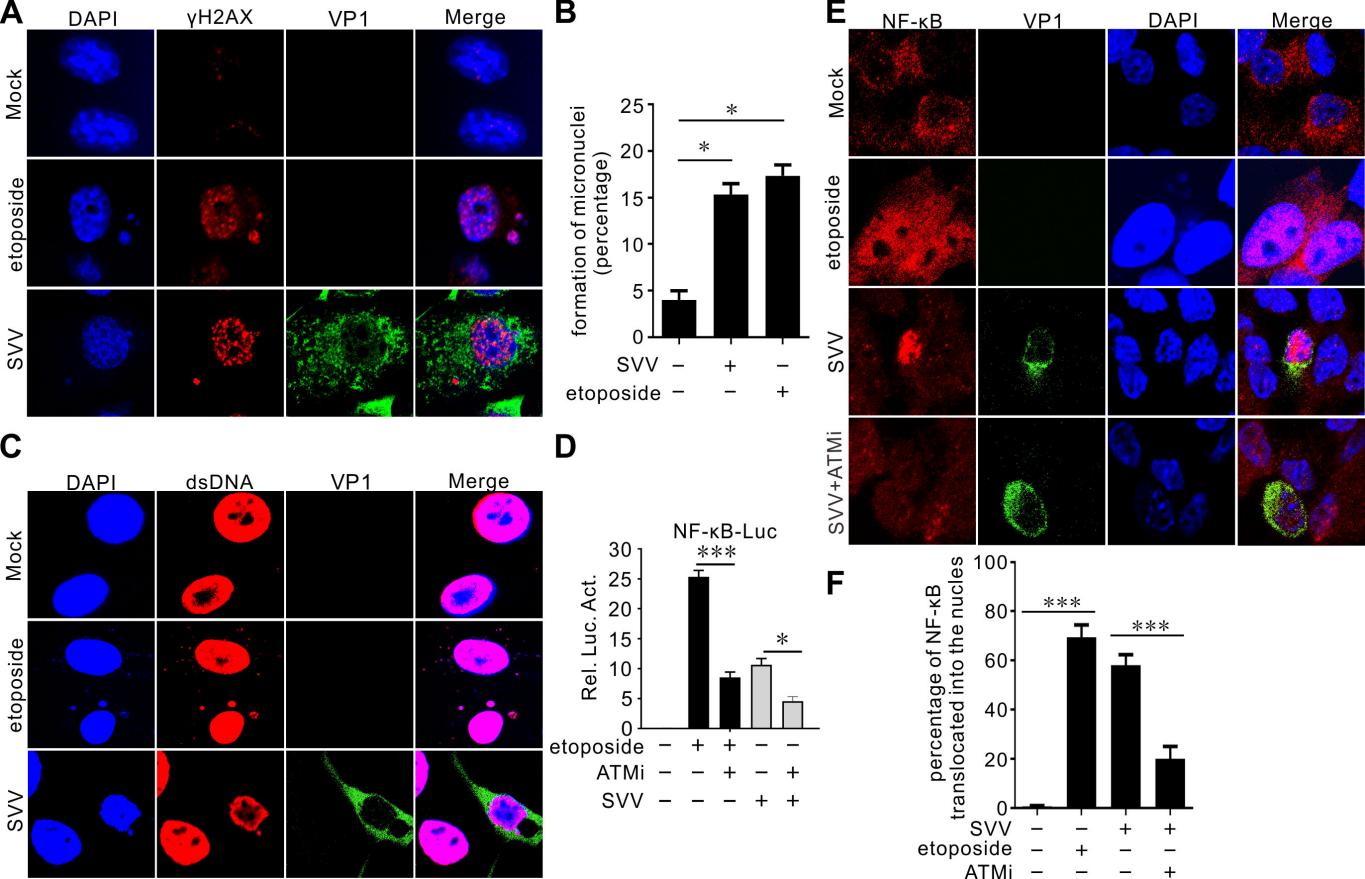
SVV-induced DDR is involved in NF-κB activity. (**A, C, and E**) BHK-21 cells infected with SVV (MOI = 1) or treated with etoposide (10 µM). Cells were stained with γH2AX antibody (red) (**A**), dsDNA (red) (**C**), NF-κB (red) (**E**), VP1 antibody (green), and DAPI (blue), then examined by confocal microscopy. (**B**) Quantification for the percentage of micronuclei formation following SVV infection or upon treatment with etoposide shown in panel **A**. Uninfected cells were used as a control. ***P < 0.001. (**D**) HEK-293T cells were transfected with NF-κB luciferase reporter plasmid for 24 h. Cells were treated with etoposide and ATMi or infected with SVV for 12 h. The samples were subjected to a dual-luciferase assay. (**F**) Quantification of the proportion of NF-κB translocation after SVV infection, treated with etoposide or ATMi, as presented in panel** E**. Uninfected cells were used as a control. ***P < 0.001, *P < 0.05.

Chromosomal instability leads to micronuclei formation, which, upon rupture, releases DNA into the cytoplasm, triggering the activation of inflammatory signaling mediated by cGAS and STING (41–43). Etoposide-induced DNA damage increased γH2AX marker levels in micronuclei (Fig. 7A and B). SVV infection induced micronuclei formation that strongly co-localized with γH2AX (Fig. 7A and B). Similarly, we observed that micronuclei were positive for cytosolic dsDNA with a monoclonal antibody against dsDNA (Fig. 7C). A previous study indicated that DNA damage can trigger inflammation, activating the cGAS-STING pathway and leading to NF-κB activation via ATM (44). Etoposide treatment or SVV infection significantly promoted NF-κB promoter activity, while the ATM-specific inhibitor (ATMi) treatment greatly attenuated etoposide-induced NF-κB activation (Fig. 7D). The addition of etoposide alone resulted in nuclear localization of NF-kB p65, whereas NF-κB p65 was mostly cytoplasmic localization (Fig. 7E and F). SVV infection greatly activated the NF-κB signaling pathway, as evidenced by nuclear translocation of the NF-κB p65 subunit, which was greatly compromised by ATMi treatment (Fig. 7E and F).

### ATM-mediated DNA DSBs promote SVV replication

**Fig 8:**
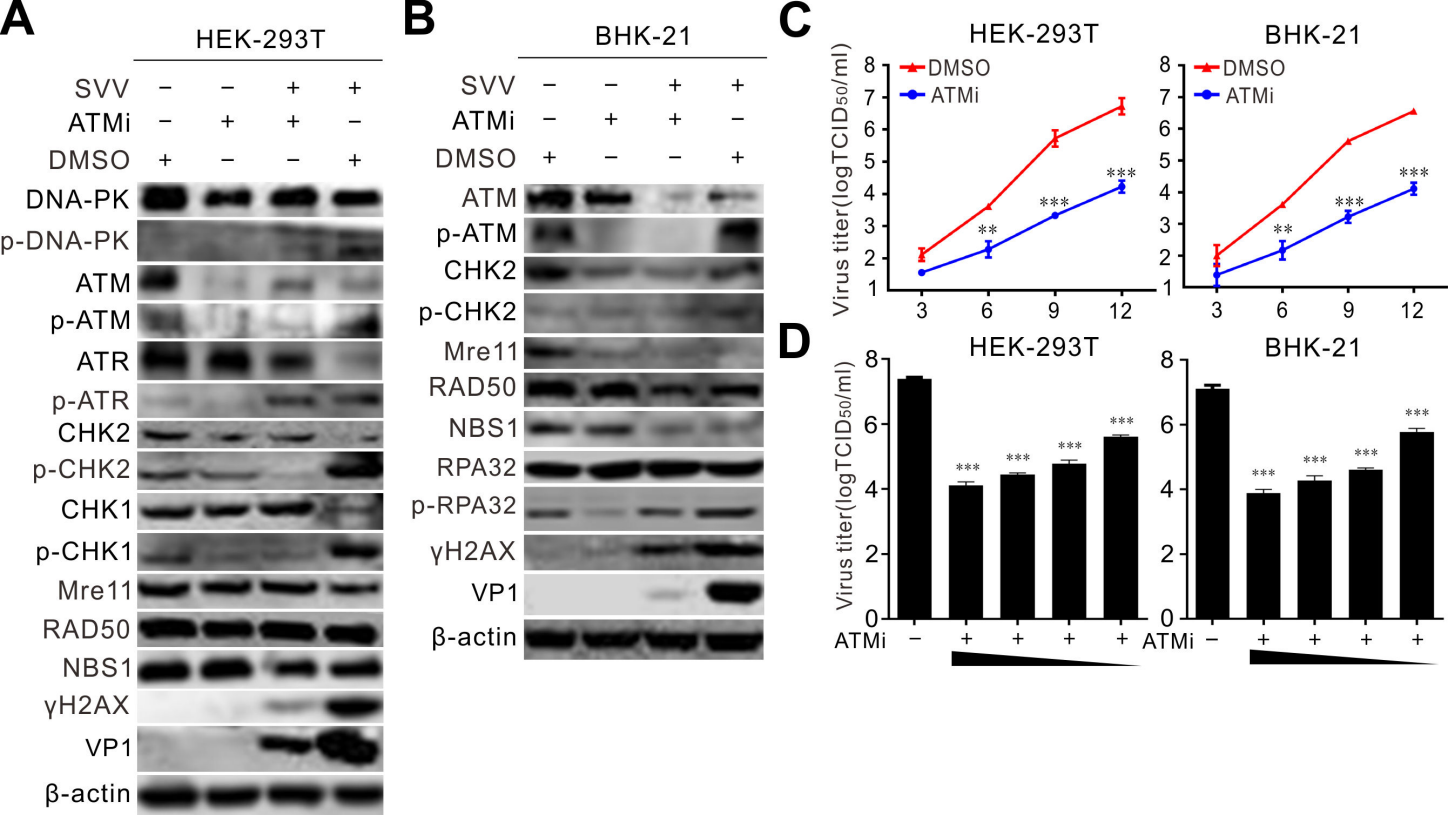
ATM-mediated DDR pathway is critical for SVV replication. (**A and B**) HEK-293T cells and BHK-21 cells infected with SVV (MOI = 5) were treated with ATMi (10 µM), respectively. The cell lysates were collected at 12 hpi and analyzed by immunoblotting with indicated antibodies. (**C**) Growth curve of ATMi-treated (10 µM) HEK-293T cells and BHK-21 cells after SVV infection (MOI = 0.5). Total viruses were titrated with the TCID_50_ assay. (**D**) Virus titers of HEK-293T cells and BHK-21 cells treated with ATMi (10 µM) with various concentrations (50, 20, 10, or 5 µM) after SVV infection (MOI = 0.5).**P < 0.01, ***P < 0.001.

DNA damage activates the type I IFN system through the cytosolic DNA sensor STING, promoting innate immunity (45). To examine the effect of the ATM-CHK2 signaling pathway on SVV replication, we inhibited ATM kinase activity using ATMi in HEK-293T and BHK-21 cells. ATMi treatment significantly inhibited the phosphorylation of ATM (Fig. 8A and B), suggesting effective blockade of ATM kinase activity. Furthermore, CHK2 phosphorylation, a downstream effector of the ATM signaling pathway, was markedly inhibited by the treatment (Fig. 8A and B). ATM inhibitors significantly dampened the protein levels of γH2AX, along with decreased p-DNA-PKcs and p-CHK1 levels (Fig. 8A and B), suggesting that ATM kinase is required for activating the ATM-CHK2 axis. Blocking ATM kinase activity greatly prevented viral VP1 production and reduced viral titers (Fig. 8A through D). These findings indicate that ATM-induced DSBs are essential for SVV replication. Collectively, these results show that the ATM-CHK2 signaling pathway plays a critical role in SVV replication.

### DNA-PK inhibition suppresses SVV replication

**Fig 9:**
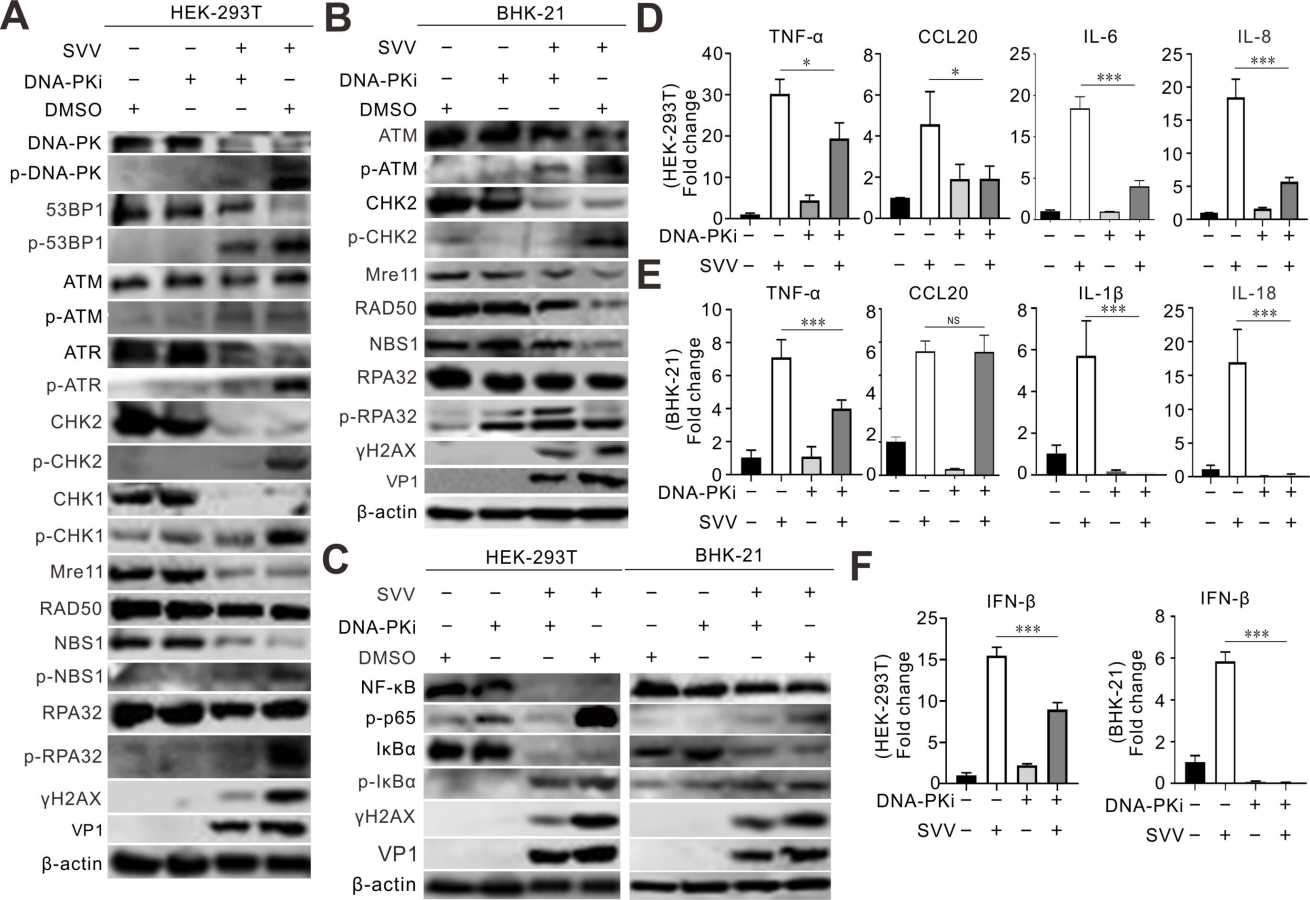
DNA-PK inhibition suppresses SVV replication. (**A–C**) HEK-293T cells and BHK-21 cells infected with SVV (MOI = 5) were treated with DNA-PKi (10 µM). The cell lysates were collected at 12 hpi and analyzed by immunoblotting with the indicated antibodies. (**D–F**) The transcriptional expression level of the indicated gene was analyzed using quantitative reverse transcription-PCR and normalized to β-actin mRNA. Error bars indicate mean ± SD from three independent infection experiments. *P < 0.05, ***P < 0.001. NS, not significant.

The DNA-PK complex, involved in DNA repair via NHEJ, has been identified as a DNA sensor that elicits the transcription of type I IFN, cytokine, and chemokine genes (46, 47). SVV infection activated DNA-PK in HEK-293T cells, which was eliminated by DNA-PKi treatment (Fig. 9A), indicating that DNA-PKi efficiently restrained the activity of DNA-PK. We examined the effects of DNA-PKi on global DDR gene expression and found that DNA-PKi inhibited the ATM-CHK2 and ATR-CHK1 pathways, suggesting that other DDR pathways were affected by the drug (Fig. 9A and B). Treatment with DNA-PKi led to a significant decrease in phosphorylation of NF-κB and IκBα elicited by SVV infection (Fig. 9C). Additionally, DNA-PKi abrogated SVV infection-associated inflammatory cytokines and interferon-β (IFN-β) induction (Fig. 9D through F). These findings suggest that DNA-PKi inhibits viral replication and probably reduces the production of inflammatory cytokines. Overall, DNA-PK is essential for SVV replication.

### DDR pathways are required for SVV infection

**Fig 10:**
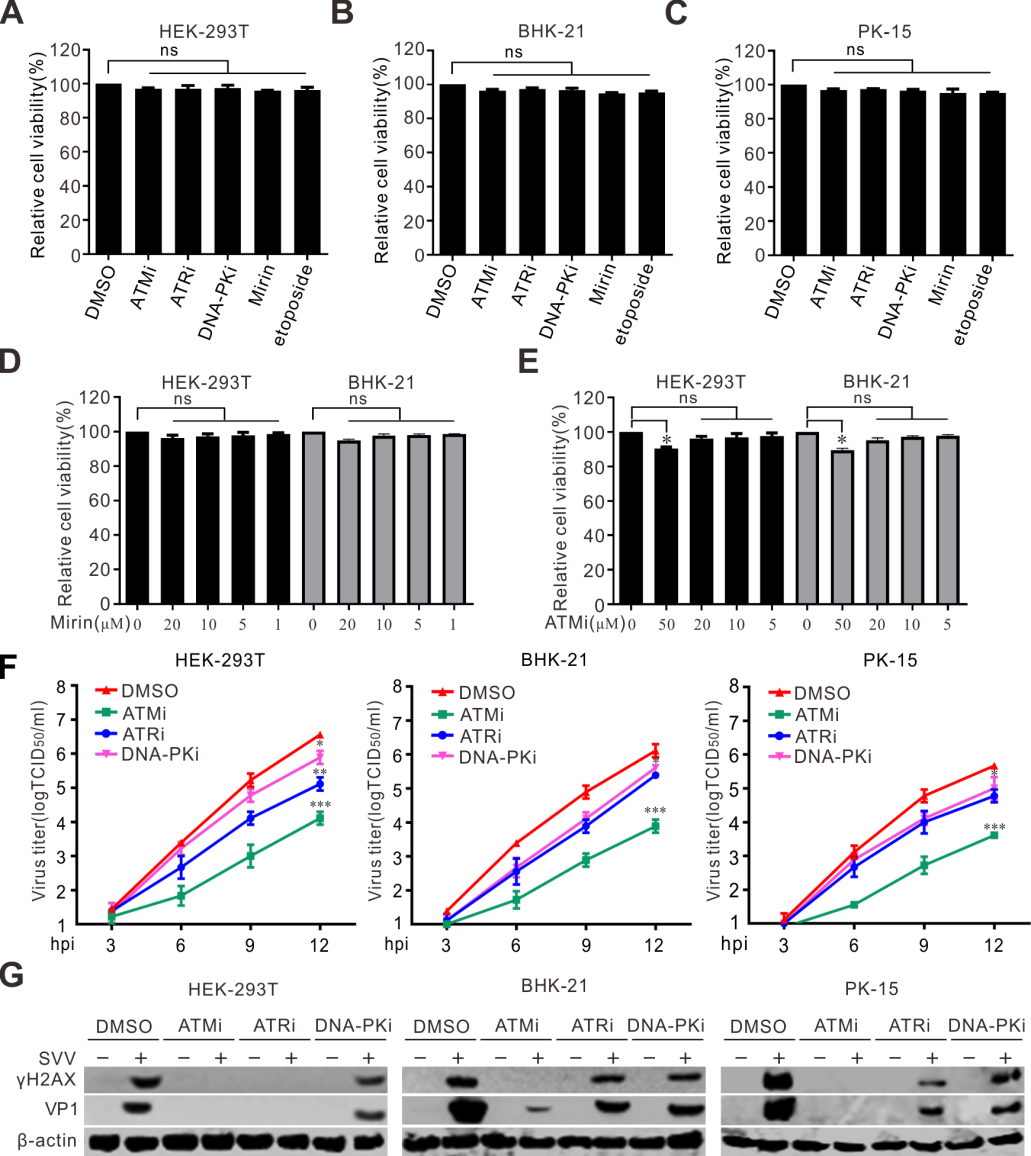
Activation of DDR is essential for efficient SVV growth. (**A–C**) The viability of HEK-293T cells, BHK-21 cells, and PK-15 cells was examined using CCK-8 assay after treatment with DMSO, ATMi (10 µM), ATRi (10 µM), DNA-PKi (10 µM), mirin (10 µM), and etoposide (10 µM) for 12 h. The error bars stand for standard deviation (SD) from three independent experiments (ns, not significant). The relative cell viability was normalized to DMSO group. (**D**) The viability of HEK-293T cells and BHK-21 cells was examined using CCK-8 assay after treatment with DMSO, mirin (20, 10, 5, 1 µM) for 12 h. The error bars stand for standard deviation (SD) from three independent experiments. The relative cell viability was normalized to DMSO group. (**E**) The viability of HEK-293T cells and BHK-21 cells was examined using CCK-8 assay after treatment with DMSO, ATMi (50, 20, 10, 5 µM) for 12 h. The error bars stand for standard deviation (SD) from three independent experiments (*, *P*0.05). The relative cell viability was normalized to DMSO group. (**F**) HEK-293T cells, BHK-21 cells, or PK-15 cells were pretreated with ATMi (10 µM), ATR (10 µM), or DNA-PKi (10 µM) and infected with SVV (MOI = 0.1). The total viruses were titrated with the TCID_50_ assay at 12 hpi. error bars indicate mean ± SD from three independent infection experiments. *P < 0.05, **P < 0.01, ***P < 0.001. (**G**) HEK-293T cells, BHK-21 cells, and PK-15 cells were pretreated with ATMi, ATR, DNA-PKi and infected with SVV for 12 h (MOI = 0.1). The cell lysates were collected and analyzed by immunoblotting with indicated antibodies. NS, not significant.

We compared the effects of DDR signaling pathways on SVV replication, including ATM, ATR, and DNA-PK, in HEK-293T, BHK-21, and PK-15 cells. Cellular toxicity was examined by Cell Counting Kit 8 assay. The results indicated that the viability of cells treated with ATMi, ATRi, DNA-PKi, mirin, or etoposide at a concentration of 5–20 μM was not significantly affected (Fig. 10A through E). Treatment with ATM, ATR, or a DNA-PK inhibitor reduced viral progeny production (Fig. 10F). Application of ATMi markedly decreased the levels of progeny virion production in the three cell types (Fig. 10F). Consistent with these findings, the application of ATMi resulted in an obvious decrease in γH2AX expression, accompanied by a reduction in viral VP1 production (Fig. 10G). Collectively, these results indicate that SVV can exploit the cellular DDR machinery for efficient replication.

## DISCUSSION

DDR is a fundamental cellular response that senses DNA damage and initiates a signaling cascade for repair and maintaining genomic integrity. Viral infection threatens host genome integrity, and numerous viruses hijack cellular DDR during their life cycles, with some species manipulating DDR components to promote replication (48–50). To maintain genome integrity, the host’s DNA repair machinery can distinguish invading or generating viral DNA from damaged DNA and can limit viral replication (48). Although the virus-modulated DDR pathway is broadly conserved, the specific mechanisms may vary with the virus type. Viruses use three main patterns to engage in DDR signaling: activation, inhibition, and degradation. In this study, by comprehensively assessing the DRR pathway during SVV replication, we found that SVV infection causes DNA damage via two distinct routes to activate cellular DDR in HEK-293T, BHK-21, and PK-15 cells.

SVV infection induced DNA damage, as evidenced by ATM-CHK2 signaling pathway activation in HEK-293T, BHK-21, and PK-15 cells (Fig. 1A through C). Further studies indicated that SVV infection induced DNA fragmentation as measured by comet assays (Fig. 1D). Additionally, the SVV-activated ATM-mediated DNA DSB response was verified by immunostaining (Fig. 2). Minute virus of canine (MVC) infection activates ATM and ATR; ATM-mediated DDR is required for inducing cytopathic effects, and the ATM pathway upstream regulator, MRN complex, facilitates the replication of the MVC genome (51). Minute virus of mice infection disables the ATR-CHK1 signaling pathway (52). Adenovirus 5 E4orf3 immobilizes the MRN complex to prevent ATR signaling (53), and adenovirus 12 E4orf6 inhibits the ATR pathway by targeting TOPBP1 for proteasomal degradation (54). We observed that ATR was activated in HEK-293T cells but not in BHK-21 and PK-15 cells (Fig. 1A through C). Human papillomaviruses (HPVs) activate ATR and suppress the transcription of inflammatory response genes, including IL-6, chemokine (C-X-C motif) ligand 2, and CXCL10 (55). HBoV1 NS1 protein induces the DDR activation of ATM, ATR, and DNA-PKcs, as reflected by NS1’s ability to induce the phosphorylation of H2AX and RPA32, alongside ATM, ATR, and DNA-PKcs activation (29). We found that SVV 2B and 2C induced host cell DNA damage, as indicated by DNA fragmentation when 2B and 2C were overexpressed or treated with etoposide (Fig. 3C and D). Additionally, SVV 2B and 2C activated the ATM, ATR, and DNA-PKcs pathways in HEK-293T cells, as shown by Western blotting (Fig. 3A) and immunofluorescence (Fig. 3B). Overexpression of individual picornaviral 2B and 2C, both membrane-targeted proteins, is known to activate ER stress and apoptosis, which may at least partially explain the DDR induced by SVV 2B and 2C (56, 57). Once DNA damage occurs, viruses modulate DDR sensors such as the MRN complex. Adenovirus 5 (Ad5) early proteins target the MRN complex for degradation but do not inhibit the global DDR signaling pathway that facilitates viral genome replication (50). MRN complexes are degraded by two mechanisms during adenoviral infection: E4-ORF3-dependent relocalization of MRN proteins and E4-ORF6/E1B-55K-dependent degradation of MRN components (58). KSHV cytosolic latency-associated nuclear antigen (LANA) recruits members of the MRN repair complex in the cytosol to inhibit NF-κB pathway activation and promote lytic replication in KSHV-infected cells (18). Similar to the KSHV LANA protein, the Ad5 E protein relocalizes and degrades components of the MRN complex (59, 60). We found that SVV infection induced the phosphorylation of NBS1 in HEK-293T cells but degraded the MRN components Mre11 and NBS1 in HEK-293T and BHK-21 cells (Fig. 4A through C). SVV 3C^pro^ contributes to the degradation of Mre11 and NBS1 (Fig. 4F). Rotavirus infection relocalizes the MRN complex to the cytoplasm through the viral NSP2 and NSP5 proteins (61), and retrovirus HTLV-1 p30 interacts with Rad50 and NBS1 to sequester and disrupt the formation of the MRN complex on DSBs foci (62). SVV infection relocalized Mre11 to the cytoplasm via viral 3C^pro^ protein (Fig. 4G), and 3C^pro^ mutants with inactivated protease activity had no impact on the distribution of Mre11 (Fig. 5C). HPV activates ATM-CHK2 but does not degrade MRN components, which is necessary for viral genome replication (63). Human herpesvirus 6B infection causes genomic instability by suppressing ATM signaling, and viral immediate-early protein 1 interacts with NBS1 to block homology-directed DNA repair (64). This indicates that the sequestration or relocalization of MRN components is a conserved strategy utilized by diverse viruses, beneficial for viral infection. MRN complex activation is incomplete, and DDR is disrupted by human cytomegalovirus infection (HCMV) (65). HCV infection of B cells increases chromosomal breaks; its core protein interacts with NBS1 and inhibits MRN complex formation, interfering with ATM activation and NHEJ (66). Bovine herpesvirus-1 VP8 protein interacts with ATM and NBS1, inhibiting NBS1 phosphorylation, disrupting the ATM/NBS1/SMC1 pathway, inhibiting DNA repair, and inducing apoptosis (67). The region of structural disorder in NBS1 interacts with the HSV-1 ICP0 protein and is required for optimal virus replication (68). We observed that NBS1 expression decreased during SVV infection, evident from the reduced abundance of NBS1 (Fig. 4A throguh C), and fluorescence intensity greatly decreased in SVV-infected cells (Fig. 4C). Additionally, SVV 3C^pro^ was responsible for endogenous NBS1 degradation (Fig. 4F). Further study showed that 3C^pro^ degraded Mre11 and NBS1 in a protease activity- and caspase pathway-dependent manner (Fig. 5A, B and D). Similarly, overexpression of 3C^pro^ induced endogenous NBS1 degradation, as indicated by the markedly decreased fluorescence intensity (Fig. 4G). The MRN complex is recruited to KSHV replication compartments to promote viral replication, and the downregulation of Mre11 and inhibition of its exonuclease activity abrogate KSHV replication (69). CHK1 loss leads to DNA damage and inflammation, and SARS-CoV-2 infection induces DNA damage through CHK1 degradation (32). ORF6 and NSP13 trigger CHK1 degradation via the proteasome and autophagy pathways, respectively (32). SVV infection induced CHK1 degradation (Fig. 1A and B), indicating that SVV 3C^pro^ causes DNA damage via CHK1 degradation.

53BP1, an ATM substrate involved in early DDR, is recruited to nuclear foci and co-localizes with H2AX upon exposure to DSBs, and 53BP1 foci formation is detected in response to etoposide (70). 53BP1 is related to the recruitment of repair proteins to DNA lesions and participates in chromosomal remodeling (70). Chlamydia infection disturbs the DDR by preventing the recruitment of p-ATM and 53BP1 to damaged sites (71). The number of 53BP1 foci significantly decreased in cells infected with SVV (Fig. 6C through E). SVV infection induced the accumulation of γH2AX foci, accompanied by co-localization with p-ATM in nuclear foci (Fig. 6H). However, in rSVV-eGFP infected BHK-21 cells, γH2AX foci accumulation was not accompanied by co-localization with 53BP1 foci (Fig. 6I). This indicates that SVV infection damages the recruitment of 53BP1 and impedes NHEJ. Unrepaired and misrepaired DSBs can cause cell death and genomic instability (72). SARS-CoV-2 N impairs 53BP1 recruitment at DSBs, hindering NHEJ repair (32). Epstein-Barr virus (EBV) tegument protein BKRF4 accumulates at DNA breaks, disrupting 53BP1 foci formation and host DDR signaling (73). The EBV ZEBRA protein induces p-ATM and p53BP1 foci (74), which interact with 53BP1, while 53BP1 depletion inhibits viral replication (75). The HIV Vpr protein modulates DDR in two independent steps: DNA damage and repression of DSB repair (76). SVV infection induces DNA damage and inhibits repair. We observed that SVV 2B and 2C contributed to the loss of 53BP1 foci, which impaired 53BP1 condensation at DSBs (Fig. 6J and K). These results suggest that SVV 2B and 2C are sufficient to reduce 53BP1 foci formation, ultimately impairing DNA repair via NHEJ.

When DDR signaling is aberrantly activated, ATM initiates multiple antiviral defense mechanisms, including activation of NF-κB, IRF1/IRF7, and upregulation of TNF-α, IL-6, and IL-8 (37, 38, 77–85). Ruptured micronuclei release DNA into the cytoplasm, triggering the inflammatory effects (41, 42). *Mycobacterium tuberculosis* inhibits DNA repair responses, triggering genomic instability and micronuclei formation that induce the cGAS-STING pathway and IFN-β production (86). SVV infection induced micronuclei formation, which was positive for γH2AX and cytosolic dsDNA (Fig. 7A through D). In almost all immortalized cell lines, there are some traces of aberrant chromosome segregation/nucleus restoration after mitosis. PRV infection triggers a non-canonical DDR-mediated NF-κB activation depending on ATM kinase, while the virus actively inhibits NF-κB-dependent gene expression (87). HCMV UL76 protein induces IL-8 expression by activating DDR (88). Consistent with previous reports, SVV triggers NF-κB activation through the DDR pathway. SVV infection results in the nuclear translocation of the NF-kB p65 subunit, and the presence of ATMi significantly inhibited SVV-induced NF-κB translocation (Fig. 7E and F).

Etoposide-induced DNA damage via ATM causes the non-canonical STING signaling to predominantly activate NF-κB rather than IRF3 (38). Inhibition of ATM attenuates the mRNA transcription of IFN-β and IL-6 and is essential for the nuclear translocation of NF-κB p65 and p65 phosphorylation (38). Treatment with ATMi significantly reduced SVV replication (Fig. 8A through D). HIV-1 accessory protein Vif counteracts Vpr-induced ATM activation and inhibits ATM-directed activation of pro-inflammatory NF-κB signaling (89). These results indicate that ATM positively regulates the NF-κB signaling pathway. DNA-PKcs are rapidly recruited to DSB damage sites and activated, and are involved in DDR and repair processes (90). DNA-PK activation is induced by adenovirus infection without requiring an MRN sensor complex (91). Additionally, coxsackievirus B5 infection causes DNA damage and activates DNA-PK and ATM (92). We found that SVV infection activated DNA-PK in HEK-293T cells but not in BHK-21 and PK-15 cells (Fig. 1A through C). HEK-293T cells stably and highly express the SV40 large T antigen that interact with multiple intracellular proteins, regulate the cell cycle, and make the cells more receptive to and capable of integrating exogenous DNA (93). However, due to the lack of the STING protein, HEK-293T cells cannot effectively activate the innate immune response (94). SVV replicates extremely faster in porcine Instituto Biologico-Rim Suino-2 (IBRS-2) cells than that in PK-15 cells. During SVV infection, the pathways related to the innate immune response were efficiently activated within PK-15 cells but not in IBRS-2 cells (95). SVV infection triggers autophagy in PK-15 and BHK-21 cells through the PERK and ATF6 pathways (96). These results demonstrated that the differences in cell characteristics possibly affect the DDR signaling pathway during SVV infection. A previous study demonstrated that a higher SVV viral load occurred at the spleen (97, 98). SVV infection induced DDR response in piglets. We observed elevated levels of γH2AX and p-ATM in SVV-infected spleens with immunohistochemical examination (Fig. 1F). These results indicated that SVV infection induced DNA damage and activated the ATM signaling pathway among different cell lines and natural host. DNA-PK inhibition suppressed SVV replication (Fig. 9A through C). The activation of NF-κB triggered by genotoxic stress differs from the traditional canonical pathway because of the shuttling of NF-κB essential modulator (NEMO) through the nucleus. DNA-PK initiates NEMO phosphorylation, enabling it to shuttle through the nucleus, subsequently leading to NF-κB activation (99). Inhibiting DNA-PK impaired SVV infection-induced NF-κB phosphorylation (Fig. 9C) and inflammatory factor transcription (Fig. 9D and E). Inhibiting ATM, ATR, and DNA-PKcs decreased SVV replication (Fig. 10F and G). Notably, ATM inhibition resulted in the largest reduction in viral titer and VP1 production (Fig. 10F and G). The results indicate that ATM, ATR, and DNA-PKcs activation promotes SVV replication, with ATM activation playing a major role.

**Fig 11:**
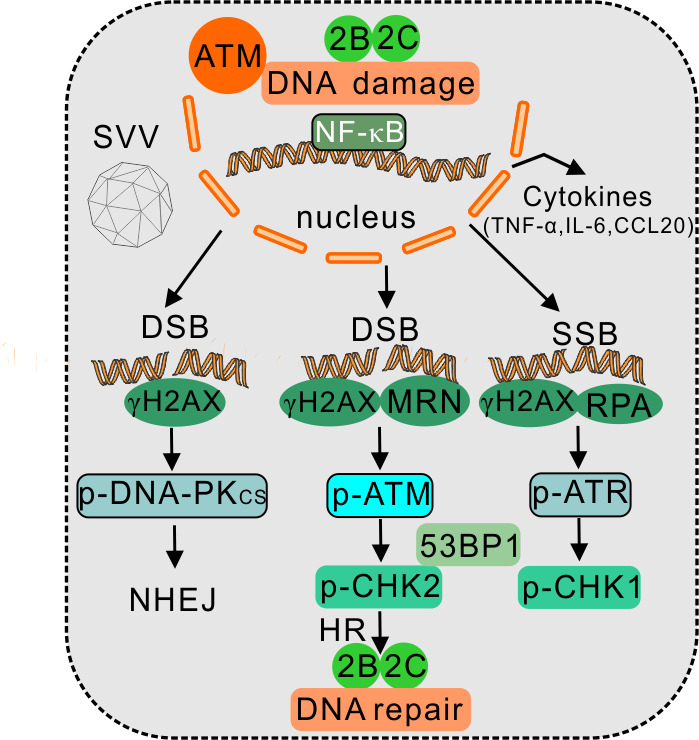
Proposed model for SVV infection induced DDR. SVV infection causes DNA damage and activates ATM-CHK2 axis, thereby triggering ATM-mediated DNA double-strand break response. Additionally, SVV infection impedes DNA repair, as it fails to induce the formation of γH2AX and 53BP1 foci. SVV 2B and 2C proteins are capable of causing DNA damage, activating DDR pathway and impairing DNA repair. The study shows that SVV employs distinct strategies to delicately adjust the DDR responses to promote viral replication.

In conclusion, the present study demonstrated that SVV infection induces DNA damage, triggers ATM-mediated DNA DSBs, and impairs DNA repair (Fig. 11). SVV 2B and 2C proteins activate DDR pathways and inhibit DNA repair. Overall, our findings provide further insights into how SVV infection manipulates the host DDR signaling pathway and the DNA damage-mediated inflammatory response for efficient replication.

## MATERIALS AND METHODS

### Cells, viruses, antibodies, and reagents

HEK-293T cells, BHK-21 cells, and PK-15 cells were grown in Dulbecco’s modified Eagle’s medium (DMEM) (Invitrogen, CA, USA) containing 10% fetal bovine serum (FBS) (Invitrogen) at 37°C with 5% CO_2_. The SVV strain CHhb17 used in our previous studies (100). rSVV-eGFP was kindly provided by Dr. Fuxiao Liu (Qingdao Agricultural University) (101). RPA32/RPA2 (2208), p-ATM (4526, Ser1981), p-53BP1 (2675, Ser1778), p-ATR (2853, Ser428), CHK1 (2360), p-CHK1 (2348, Ser345), p-CHK2 (2197, Thr68), CHK2 (6334), Mre11 (4847), Rad50 (3427), p-p95/NBS1 (3001, Ser343), p95/NBS1 (14956), p-IkBα (2859, Ser32), IkBα (9247), p-NF-κB p65 (3033, Ser536), and NF-κB p65 (6956) were purchased from Cell Signaling Technology (Danvers, MA, USA). p-DNA-PKcs (phospho S2056), p-RPA32/RPA2 (phospho S33), anti-ATR (2B5), and anti-dsDNA mouse monoclonal (ab27156) were purchased from Abcam (Cambridge, Cambridgeshire, UK). 53BP1 (A3859), DNA-PKcs (A20837), and ATM (A19650) were purchased from ABclonal (Wuhan, China). p-Histone H2AX (sc-517348, Ser139) and p-ATM (sc-47739) were purchased from Santa Cruz (Dallas, TX, USA). Horseradish peroxidase (HRP)-conjugated goat anti-rabbit IgG (H + L) (1706515, Bio-Rad), HRP-conjugated goat anti-mouse IgG (H + L) (1706516, Bio-Rad), ﬂuorescein isothiocyanate-conjugated goat anti-mouse IgG (H + L) (F0257, Sigma), Alexa-568-conjugated goat anti-rabbit IgG (H + L) (11011, Invitrogen), and mouse anti-VP1 monoclonal antibody have been used in our previous studies (100). InSolution ATM kinase inhibitor (ATMi, 118502), ATR kinase inhibitor (ATRi, 118510), and DNA-PK inhibitor II (DNA-PKi, 260960) were obtained from Merck (Rahway, NJ, USA). MG132 (S2619), Z-VAD-FMK (S7023), bafilomycin A1 (S1413), chloroquine (CQ, S6999), and 3-methyladenine (3-MA, S2767) were purchased from Selleck Chemicals (Shanghai, China).

### Plasmid construction

GFP-tagged SVV structural and non-structural protein plasmids, single-point mutant plasmids GFP-3C^H48A^ and GFP-3C^C160A^, and double mutant plasmid GFP-3C^[DM]^ (H48A and C160A double mutants) have been used in our previous studies (7, 10). The Mre11 and NBS1 gene were synthesized from RuiBiotech Biotechnology Co., Ltd. (Beijing, China) and reconstructed into pCMV-HA vector (Clontech, 631604) and p3 ×FLAG-CMV-10 vector (E4401, Sigma), respectively, by using one-step DNA assembly kit (D0204P; Lablead, Beijing, China).

### Western blotting

Cells were harvested and lysed using lysis buffer (0.5% NP-40, 50 mM Tris, 0.5 mM EDTA, and 150 mM NaCl) containing phenylmethanesulfonyl fluoride. Cell lysates were subjected to sodium dodecyl sulfate-polyacrylamide gel electrophoresis and transferred onto a nitrocellulose membrane (66485; Pall, Florida, USA), then were blocked in 5% non-fat dry milk in phosphate-buffered saline (PBS) for 2 h (room temperature [RT]). Membranes were incubated with appropriate primary antibodies overnight at 4°C with a rotation, then washed with PBS containing Tween-20 (PBST) and incubated with secondary antibody for 1 h (RT). The membranes were washed with PBST and incubated with enhanced chemiluminescence (E1070; Lablead, China).

### Comet assays

A comet assay was performed using the DNA damage comet assay kit (C2041M, Beyotime), following the manufacturer’s alkaline comet assay protocol. Briefly, cells were harvested, washed once, and resuspended in PBS. Cells were then mixed with comet agarose (1:3 cell-to-agarose ratio) and solidified on a comet slide. Gel electrophoresis was performed by transferring the slide to an electrophoresis apparatus filled with electrophoresis solution. Slides were then subjected to 25 V for 20–30 min at constant 300 mA. The slides were immersed in cold lysing solution for 2 h at 4°C. After electrophoresis, the slides were neutralized and incubated in 0.4 mol/L Tris-HCl buffer for 5 min. Then, the slides were washed with ethanol; the colloidal surface moisture was dried (RT); and then ethidium bromide was added to the colloidal surface and covered with the cover glass for 20 min. Comets were imaged on a fluorescence microscope.

### Indirect immunofluorescence assay

The cells were seeded on coverslips in 24-well plates and fixed with 4% paraformaldehyde for 10 min at room temperature (RT), subsequently permeabilized with 2% bovine serum albumin (BSA) containing 0.1% Triton X-100 for 10 min (RT), then blocked with 2% BSA for 30 min (RT). Samples were incubated with appropriate primary antibodies overnight at 4°C. After washing with PBS, the cells were probed with secondary antibodies for 1 h (RT). Images were captured under a Nikon Al confocal microscope (Tokyo, Japan).

### TCID_50_ assay

BHK-21 cells were infected with SVV at the multiplicity of infection indicated. After incubation with SVV for 1 h at 37°C, the cells were washed three times with DMEM and supplemented with fresh culture medium containing 2% FBS. The cultured medium and cells were collected at the indicated times after SVV infection, then samples were titrated on BHK-21 cells using limiting dilution assay by using TCID_50_ assay.

### Quantitative reverse transcription-PCR (RT-qPCR)

Total RNA was extracted using FastPure Cell/Tissue Total RNA Isolation (Vazyme, RC101-01). One microgram of RNA was reverse transcribed into cDNA using First-Strand Synthesis Master Mix (F0202, Lablead). RT-qPCR was conducted using Realab Green PCR mix (R0202, Lablead). The formula 2^−△△CT^ was adopted to calculate the relative expression fold change of the target gene. The RT-qPCR targeted a conserved genomic region within the 3D gene of SVV. The amount of viral RNA detected in samples was expressed as log10 genome copy number per milliliter. The primer sequences are listed in Table 1.

**TABLE 1:** Primers used in this study

Primer^*a*^	Sequence (5’−3’)
Mre11-F	TGGCCATGGAGGCCCGAATTCGGATGAGTACTGCAGATGCACTTG
Mre11-R	GATCCCCGCGGCCGCGGTACCTTATCTTCTATTTCTTCTTAAA
NBS1-F	CAAGCTTGCGGCCGCGAATTCAATGTGGAAACTGCTGCCCGCCG
NBS1-R	CCTCTAGAGTCGACTGGTACCTTATCTTCTCCTTTTTAAATAA
Q-IL-6-BHK-F	CGCAAGAGACTTCCATCCACT
Q-IL-6-BHK-R	TGAAGTCTCCTCTCCGGACTT
Q-TNF-BHK-F	CAACCCTATCATCGGCTCCA
Q-TNF-BHK-R	TAAACCAGGTACAGCCCGTC
Q-CCL20-BHK-F	GTCAGAAGCAGCAAGCAACTTT
Q-CCL20-BHK-R	TTTGGATCAGCACACACGGAT
Q-IL-1β-BHK-F	ACAGAAATGCCTCGTGCTGT
Q-IL-1β-BHK-R	GTGGGCGTGTCACCTTTCAT
Q-IL-18-BHK-F	TGGAGACCTGGAATCAGACGA
Q-IL-18-BHK-R	ACAGAGAGGGTTACAGGCAGT
Q-IFNβ-BHK-F	GAGGAACTTGAGGCCAGACAA
Q-IFNβ-BHK-R	CCATCTGTTCTGGGTGGTTCA
Q-IL-6-human-F	TTCGGTCCAGTTGCCTTCTC
Q-IL-6-human-R	CTGAGATGCCGTCGAGGATG
Q-IL-8-human-F	GAGAGTGATTGAGAGTGGACCAC
Q-IL-8-human-R	CACAACCCTCTGCACCCAGTTT
Q-TNF-human-F	GCTGCACTTTGGAGTGATCG
Q-TNF-human-R	TCACTCGGGGTTCGAGAAGA
Q-CCL20-human-F	GCGAATCAGAAGCAGCAAGCA
Q-CCL20-human-R	GCCGTGTGAAGCCCACAATAA
Q-IFNβ-human-F	TCTTTCCATGAGCTACAACTTGCT
Q-IFNβ-human-R	GCAGTATTCAAGCCTCCCATTC
Q-SVV-3D-F	AACGTCCCTTATCAACCTCCTT
Q-SVV-3D-R	ATCGACGAGATCAGTACGTCGA

^*a*^ F denotes forward PCR primer; R denotes reverse PCR primer.

### Luciferase reporter assay

HEK-293T cells were seeded in 24-well plates and transfected with 100 ng of NF-κB reporter plasmid and 20 ng of pRL-TK plasmid. At 24 h post-transfection, cells were infected or treated with SVV and etoposide for 12 h. The samples were examined with luciferase activity assays using the dual-luciferase reporter assay kit (Promega) following the manufacturer’s instructions with Renilla luciferase activity as a reference.

### Animal trials

Thirty-day-old specific-pathogen-free piglets were purchased from Beijing Center for SPF Swine Breeding and Management and verified to be negative for porcine reproductive and respiratory syndrome virus, porcine circovirus type 2, pseudorabies virus (PRV), classic swine fever virus, and SVV by reverse transcription-PCR or PCR. Piglets (*n* = 3) were inoculated intranasally with SVV strain CHhb17. The control group (mock-infected group, *n* = 3) was inoculated with cell culture supernatant. Spleen tissue samples were collected during necropsy at 10 days post-infection, fixed with 4% paraformaldehyde solution for 48 h, and then subjected to immunohistochemistry.

### Statistical analysis

Statistical analysis was processed using GraphPad Prism. Results were displayed as means ± standard deviations of three independent tests. The error bars represent the standard deviation. *P < 0.05, **P < 0.01, and ***P < 0.001 were considered statistically significant.

## Data Availability

The data supporting this study’s findings are available from the corresponding author upon reasonable request.
